# Profiling and Association over Time between Disability and Pain Features in Patients with Chronic Nonspecific Neck Pain: A Longitudinal Study

**DOI:** 10.3390/jcm11051346

**Published:** 2022-02-28

**Authors:** Gorka Ortego, Enrique Lluch, Pablo Herrero, Shellie Ann Boudreau, Victor Doménech-García

**Affiliations:** 1 Faculty of Health Sciences, Universidad San Jorge, Campus Universitario, Autov. A23 km 299, 50830 Villanueva de Gállego, Zaragoza, Spain; gortego@usj.es (G.O.); vdomenech@usj.es (V.D.-G.); 2 Department of Physiotherapy, University of Valencia, 46010 Valencia, Spain; enrique.lluch@uv.es; 3 Physiotherapy in Motion, Multi-Speciality Research Group (PTinMOTION), Department of Physiotherapy, University of Valencia, 46010 Valencia, Spain; 4 Brussels “Pain in Motion” International Research Group, Departments of Human Physiology and Rehabilitation Sciences, Vrije Universiteit Brussels, 1050 Ixelles, Belgium; 5 Department of Physiatry and Nursing, Faculty of Health Sciences, IIS Aragon, University of Zaragoza, 50009 Zaragoza, Spain; 6 Center for Neuroplasticity and Pain (CNAP), SMI, Department of Health Science and Technology, Faculty of Medicine, Aalborg University, 9220 Aalborg, Denmark; sboudreau@hst.aau.dk

**Keywords:** disability, chronic nonspecific neck pain, pain sensitivity

## Abstract

Objectives: To longitudinally investigate the relationships between neck/arm disability and pain profile measures in individuals with chronic nonspecific neck pain (NSNP) at baseline, one month, and six months after a standardized physiotherapy intervention. A secondary aim was to compare pain sensitivity of individuals with chronic NSNP at baseline to healthy controls. Methods: A total of sixty-eight individuals with chronic NSNP and healthy controls were recruited. Neck disability index (NDI), the 11-item disabilities of the arm, shoulder, and hand questionnaire (QuickDASH), temporal summation (TS), pressure pain thresholds (PPTs), pain intensity and pain extent were assessed in individuals with chronic NSNP. For the cross-sectional assessment, TS and PPTs were compared to healthy controls. Results: After following a standardized physiotherapy intervention, local and distal PPTs to the neck region decreased at one and six month follow-ups, respectively. Pain extent decreased at one and six months. Furthermore, a positive correlation between neck/arm disability and pain intensity was found at baseline, whereas moderate positive correlations (e.g., between NDI and pain extent) at baseline, one and six month follow-ups and negative correlations at six months (e.g., between arm disability and PPTs) were found. Discussion: Overall, these findings indicate that pain sensitivity can worsen following treatment despite reduced pain extent and unchanged neck disability and pain intensity scores over a six-month period in individuals with chronic NSNP.

## 1. Introduction

Neck pain is a common musculoskeletal disorder and a leading cause of disability, work loss, and health care costs worldwide [1]. Approximately one-third to one-half of all adults will experience neck pain during one year [2]. In most cases, neck pain resolves within 3–6 months, but 14% of patients develop chronic symptoms after a first pain episode [3]. Nonspecific neck pain (NSNP) refers to neck pain (with or without radiation) without evidence of a specific systemic disease or pathology as the underlying cause [4]. NSNP is a heterogeneous disorder where central and peripheral mechanisms are influenced by multiple factors, such as pain sensitivity or pain extent. In addition to symptoms in the neck region, NSNP is often associated with upper limb disability [5].

To date, outcomes for physiotherapy interventions matched to unifactorial neck pain subgroups (e.g., patients with restricted cervical movement) remains inconclusive, probably due to the heterogeneity of the study population [6]. In other words, unifactorial subgrouping underestimates the complexity of NSNP presentations. Thus, profiling people with NSNP across multiple factors or features, including pain sensitivity, may facilitate tailored interventions and improve clinical outcomes, such as pain reduction and function enhancement [7].

Quantitative sensory testing (QST) is a non-invasive method used for assessing pain sensitivity. These assessments provide insights about the underlying pathophysiological mechanisms, assessment, classification, and prognosis of musculoskeletal pain conditions [8,9]. Regarding neck pain, individuals with chronic whiplash associated disorders have demonstrated lower pressure pain thresholds (PPTs) [10]. Those with radiating neck-arm pain show reduced heat pain thresholds at distant sites from the primary site of injury [11]. High pain sensitivity at distant sites from the painful region has also been found by a recent metanalysis in individuals with nontraumatic neck pain [12]. These results are indicative of altered central pain processing in these populations.

Temporal summation (TS) is a progressive increase in pain intensity with repeated painful stimulation [13] and is an otherwise healthy response to a painful input facilitated at the spinal cord level [14]. Previous studies have demonstrated enhanced TS in individuals with chronic NSNP compared to controls [15,16], and in individuals with radiating neck pain compared with localized neck pain [11]. However, a recent study found no correlation between TS and PPTs [16], making the relationship less clear in individuals with chronic NSNP.

It has been found that high pain sensitivity evidenced by QST is more common in individuals with higher levels of disability and neck pain [17,18]. However, the relationship between pain sensitivity, disability and pain level is less clear [19]. Previous cross-sectional studies suggest that neck disability is not related to pain sensitivity [20,21,22] although longitudinal studies are necessary. Pain in several body regions is common when the pain becomes chronic [23]. In response to exercise-induced experimental pain, application of a painful stimulus extends further from the primary stimulus location in individuals with a recovered painful trauma (i.e., fracture) [24], chronic low back pain [25], and chronic neck pain [26] as compared to healthy controls. Cumulatively, these findings suggest that persistent changes in the central processing of pain occur in recovered pain-free individuals and those with ongoing pain.

The time course of pain sensitivity development (i.e., the proneness to react to either pathological or experimental stimuli) along with natural history and after physiotherapy treatment represent less explored research topics in individuals with chronic NSNP. To date, QST profiling studies are cross-sectional and cluster individuals with chronic NSNP according to pain sensitivity, pain extent, and clinical features [27]. However, cross-sectional studies do not account for potential changes in QST-profiles over time. Thus, to better understand the time course of pain sensitivity change, it is important to start assessing pain sensitivity over different time points along the course of physiotherapy interventions.

This study aimed to investigate the relationships between neck/arm disability, pain sensitivity, pain intensity and pain extent before (baseline), at one and six months follow up after a standardized physiotherapy intervention in individuals with chronic NSNP. A secondary aim was to profile and compare the pain sensitivity of individuals with chronic NSNP at baseline to healthy controls. It was hypothesized that neck/arm disability would be positively associated with pain sensitivity, pain intensity, and pain extent over time. A secondary hypothesis is that pain sensitivity is higher in individuals with chronic NSNP compared to healthy controls.

## 2. Materials and Methods

### 2.1. Study Design

This longitudinal study assessed neck/arm disability, pain sensitivity, pain intensity, pain duration, and pain extent in individuals with chronic NSNP and age- and gender-matched healthy controls. The study was conducted from May 2020 to January 2021 in accordance with the STROBE statement [28]. Before starting the study, all individuals read and signed an informed consent. The local research ethics committee approved the study (record no. 19/17), and all procedures were conducted in accordance with the Helsinki Declaration. The study was registered on ClinicalTrials.gov (NCT04330573).

### 2.2. Setting and Evaluators

Individuals with chronic NSNP were recruited by referral from a public hospital rehabilitation service, while healthy controls were recruited through advertisements placed at the hospital and word of mouth information. One assessor with over 15 years of clinical experience (GO) performed all the outcome measures in both groups. Three independent physiotherapists performed the conservative physiotherapy treatment. Healthy controls were assessed at baseline for the cross-sectional study, whereas individuals with chronic NSNP were assessed at baseline and at 1-month and 6-month follow-ups for the longitudinal study. All individuals with chronic NSNP received a 1-month conservative physiotherapy treatment based on daily 45-min sessions of therapeutic exercise for the cervical spine (motor control exercises) and transcutaneous electrical nerve stimulation, implemented at public hospital, which is the standardized treatment for patients with chronic NSNP. It is important to remark that the physiotherapy intervention was not part of the study design, and therefore it was included a cohort of patients who received usual care. The intention was to evaluate the participants in their real context without interfering with their treatment. All treatments that participants received were similar. From the 1-month to 6-month follow-up, none of the participants received any physiotherapy treatment.

### 2.3. Participants

Inclusion criteria for individuals with chronic NSNP were: (1) aged between 18 and 65 years; (2) being able to understand, write, and speak in Spanish; (3) having suffered neck pain for at least 12 weeks; (4) having pain localized in the neck region with or without pain radiation [4,29]; (5) negative Spurling test; (6) a score < 12 on the Self-Administered Leeds Assessment of Neuropathic Symptoms and Signs (S-LANSS) [30]; (7) negative Upper Limb Neurodynamic Test 1 (ULNT1) [31]; and (8) a score < 12 on the PainDetect [32]. Criteria 5–8 were considered in order to only include individuals with NSNP without a predominant neuropathic pain mechanism.

Individuals with chronic NSNP were excluded from the study if any of the following criteria were present: (1) presence of red flags or serious pathology as reported in the medical history (i.e., tumor, fracture, metabolic diseases, rheumatoid arthritis, osteoporosis); (2) cervical radiculopathy [33]; (3) fibromyalgia; (4) previous neck surgery; (5) cervical myelopathy; and (6) currently undergoing any neck pain treatment or having received neck pain treatment in the previous three months.

### 2.4. Outcome Measures

The primary outcome measures of this study were neck/arm pain and disability measured with the neck disability index (NDI) and the 11-item disabilities of the arm, shoulder, and hand questionnaire (QuickDASH). The secondary outcome measures were pain sensitivity (PPTs, TS), pain intensity and pain extent. Pain intensity and pain extent were only measured in the chronic NSNP group. Sociodemographic information, including sex, age, height, and weight, was also collected in both the chronic NSNP and healthy control groups.

### 2.5. Neck/Arm Disability

The Spanish version of the Neck Disability Index (NDI) and QuickDASH were used in this study for assessing neck pain and disability. The NDI is a patient-reported outcome measure which includes 10 items with each item rating from 0 to 5 (total score: 0–50) [34]. The test–retest reliability is adequate (ICC 0.97) and it has been validated for the Spanish language [35].

The QuickDASH is a shortened version of the DASH questionnaire and assesses upper extremity disability in individuals with neck pain [36,37]. Instead of 30 items, the QuickDASH includes 11 items that measure physical function and symptoms. The QuickDASH is an acceptable and valid questionnaire with high internal consistency (Cronbach’s α = 0.92–0.95) and test–retest reliability (ICC = 0.90–0.94) [38,39].

### 2.6. Pain Intensity

Individuals rated self-perceived neck pain on a visual analogue scale (VAS), displayed as a 10-cm line with anchors 0 (“no pain”) and 10 (“worst possible pain”). The VAS is a reliable and valid tool for measuring pain [40,41]. For each individual, the maximum VAS score reported during the last 30 days and the perceived mean VAS score during the last 30 days were assessed in each session [42,43].

### 2.7. Pain Sensitivity

#### 2.7.1. Pressure Pain Thresholds (PPTs)

A handheld pressure algometer (Somedic, Hörby, Sweden) with a 1-cm^2^ probe was used to record PPTs unilaterally at three locations: (1) the upper trapezius of the most symptomatic side (2) bilaterally at three locations in the infraspinatus muscle, and (3) unilaterally at one location in the gastrocnemius muscle on the same side as the most symptomatic side for the trapezius muscle.

The infraspinatus muscle sites were defined as follows: (1) immediately lateral to the midpoint of the medial border of the scapula; (2) at the cut-point of three lines coming from the medial point of the scapular spine, the inferior angle of the scapula, and the midpoint of the medial border of the scapula; and (3) 2 cm over the inferior angle of the scapula [44]. PPTs were measured at three sites of the infraspinatus in order to account for the heterogeneity in the distribution of pressure pain sensitivity in this muscle [45]. The upper trapezius muscle sites were: (1) 2 cm lateral to the spinous process of C7; (2) midway between the spinous process of C7 and the acromion [21], and (3) 2 cm lateral to the acromion [46]. The gastrocnemius site was located on the distal third on a line connecting the popliteal line with the calcaneus [44]. This gastrocnemius site was chosen as a distant location which could provide information about widespread pain sensitivity changes [44].

Individuals were instructed to indicate when the pressure changed from a non-painful to a painful sensation, which corresponds with the definition of PPT [47]. Three PPT measurements were taken at each location, and the mean was used for analysis. Different studies have shown the validity and reliability of measuring PPTs among assessors, even when these latter have no previous experience with the procedure [48,49]. Moreover, PPTs measurement has shown satisfactory psychometric properties in individuals with and without chronic neck pain [50].

#### 2.7.2. Temporal Summation (TS)

TS was assessed according to the protocol described by Rolke et al. [51]. A weighted pinprick stimulator (256 mN) with a contact area of 2 mm was used to apply a train of 10 stimuli at 1 Hz frequency. Participants were asked to rate the pain experienced after the first and tenth stimulus on a 0–10 numeric rating scale. The wind-up ratio was calculated by subtracting participants’ 1st pinprick pain rating from the 10th one. Higher (positive) wind-up ratio scores indicate an enhanced TS [52,53]. TS has shown acceptable test–retest reliability (within session) (ICC 0.69–0.86) [54].

### 2.8. Pain Extent

Individuals were asked to draw the area of pain where they experienced pain most frequently during the last 30 days on a tablet using a digital body-mapping software (NavigatePain©, Aalborg University, Aalborg, Denmark). The pain area was extracted and expressed in pixels. Pain drawings measurement has been shown to be reliable in different pain populations, including people with neck pain [55,56,57,58]. Digital pain drawings are reliable, valid, and therefore comparable to paper drawings [59].

### 2.9. Procedure

All participants were assessed within a single session both at baseline (for the chronic NSNP and healthy control group) and at 1-month and 6-month follow-ups (only for the chronic NSNP group). Sociodemographic information and pain drawings were first collected. Subsequently, pain sensitivity measurements were always performed in the same order (i.e., PPTs followed by TS).

### 2.10. Sample Size Calculation

Sample size was calculated using a sample calculation software (Mallinckrodt General Clinical Research Center, Boston, MA, USA) based on the NDI and QuickDASH as the primary outcome measures. This calculation revealed a minimum sample size of 13 individuals per group for a power of 0.9 using NDI (minimum detectable change 8.4; standard deviation 8.1) [60] and 25 individuals per group for a power of >0.8 calculated with QuickDASH (minimum detectable change 11; standard deviation 18.6). Considering the biggest sample size (i.e., with QuickDASH) and 30% dropout rate, the sample size calculation was increased to 34 individuals per group.

### 2.11. Statistical Analysis

Data were analyzed using SPSS version 21.0 (SPSS, Inc., Chicago, IL, USA). Results are expressed as mean and standard deviation (SD), considering the 95% CI. The Kolmogorov–Smirnov test was used to analyze the normal distribution of the data. Quantitative data with a normal distribution were analyzed with parametric tests. In the cross-sectional part of this study, an independent *t*-test was performed to compare the outcome measures (PPTs and TS) between the chronic NSNP and healthy control groups. Effect sizes were calculated through Cohen’s d according to the formula d = mean difference/SD. Effect sizes are small if d ≤ 0.2, medium if d ≥ 0.2 or d ≤ 0.8, and large if d > 0.8 [61]. Data loss at 1-month and 6-month follow-ups was handled by the last observation carried forward method.

A repeated-measures analysis of variance (ANOVA) with Bonferroni post-hoc test was performed in the chronic NSNP group to compare all the outcome measures between baseline, 1-month and 6-month intervals. Moreover, variables were calculated in the chronic NSNP group for changes in NDI and QuickDASH, PPTs, TS, pain intensity (maximum/mean) and pain extent. In particular, 1-month and 6-month results for all those variables were subtracted from baseline. Positive change scores for neck/arm disability, TS, pain intensity, and pain extent, and negative change scores for PPTs were considered as indicative of improvement in participants’ clinical condition. The influence of pain duration at baseline on the remaining outcome measures was analysed as a co-variable with an analysis of covariance (ANCOVA).

Correlations between neck/arm pain and disability measured with the QuickDASH and NDI and the rest of outcome measures (pain sensitivity, pain intensity, pain duration and pain extent) in the chronic NSNP group at baseline, 1 month, and 6 months were estimated using Pearson’s correlation coefficient. Correlations between neck/arm pain and disability changes and the rest of outcome measures over time were also estimated using the Pearson’s correlation coefficient. For all statistical analyses, differences were considered significant at *p* < 0.05.

## 3. Results

**Figure 1:**
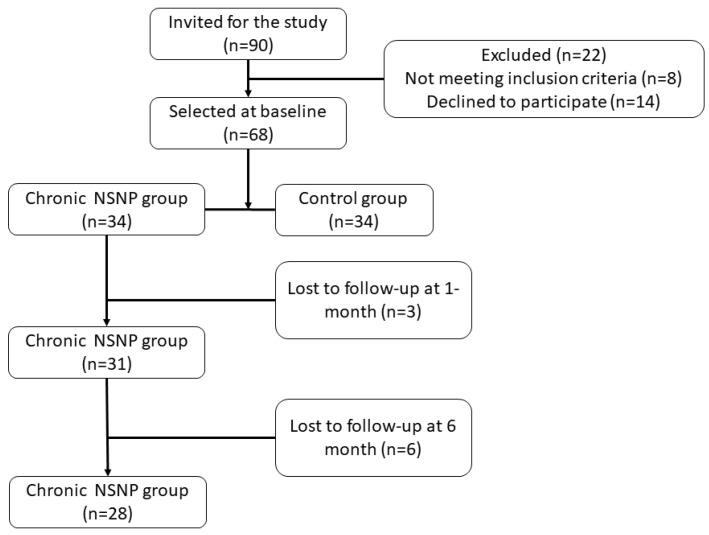
Flowchart of participants including their follow-up. Abbreviations: Nonspecific neck pain (NSNP).

A total of 68 individuals participated in the study, resulting in 34 individuals in the chronic NSNP and healthy control groups, as shown in Figure 1. Demographics for each group were similar (*p* > 0.05) as listed in Table 1. Nine individuals from the chronic NSNP group dropped-out in the follow-up (three at one month and six at six months).

### 3.1. Longitudinal Assessments of Neck/Arm Disability, Pain Sensitivity, Pain Intensity and Pain Extent in the Chronic NSNP Group (Longitudinal Study)

**Figure 2:**
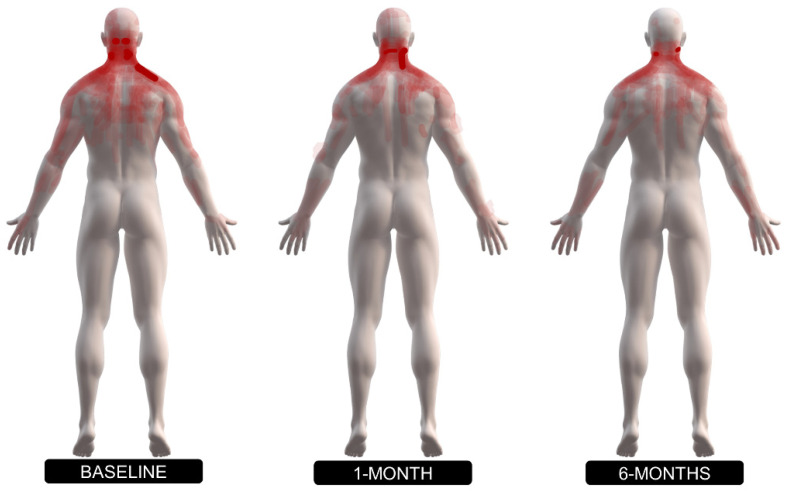
Superimposed body diagrams of pain extent and pain distribution of individuals with chronic nonspecific neck pain (NSNP) at baseline, 1 and 6-month follow-ups.

**Figure 3:**
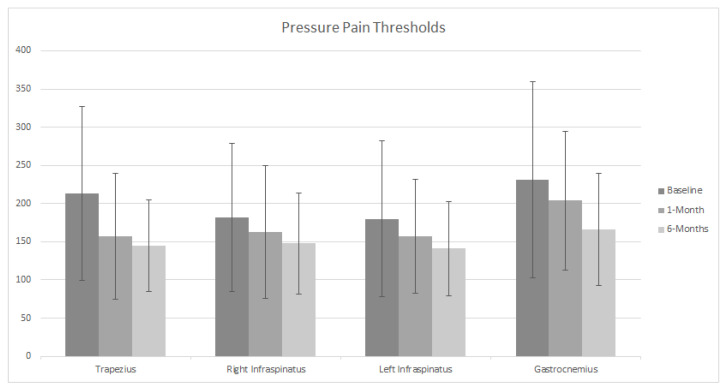
Pressure pain threshold (PPT) at baseline (n = 34), 1-month (n = 34) and 6-month (n = 34) in individuals with chronic nonspecific neck pain (NSNP) undergoing a 1-month conventional physiotherapy treatment. Data are presented for Trapezius, right Infraspinatus, left Infraspinatus and Gastrocnemius muscles in individuals with chronic NSNP. Data are presented as mean ± standard deviation.

Neck/arm pain and disability, pain sensitivity, pain intensity, and pain extent in the chronic NSNP group at baseline, one month, and six month follow-ups, are summarized in Table 2. The pain area was mainly distributed through the neck and one or two upper extremities at baseline, one month, and six month follow-ups (Figure 2). Mean PPTs and standard deviation are illustrated in Figure 3.

Neck/arm disability, TS, and pain intensity remained unchanged over time in the chronic NSNP group (Table 2). However, PPTs of the upper trapezius muscle decreased at one- and six-month follow-up compared to baseline (Trapezius 1: F_(1.421, 38.372)_ = 13.7. *p* = 0.001, *p* = 0.002. η^2^ = 0.33; Trapezius 2: F_(1.272, 34.354)_ = 13.48. *p* = 0.00, *p* = 0.005. η^2^ = 0.33; Trapezius 3: F_(1.451, 39.184)_ = 9.51. *p* = 0.007, *p* = 0.008. η^2^ = 0.26) (Table 2)_._ PPTs measured at gastrocnemius (F_(1.271, 34.325)_ = 6.37. *p* = 0.003. η^2^ = 0.19) and ‘left infraspinatus 2’ (F_(1.305, 35.231)_ = 5.6. *p* = 0.04. η^2^ = 0.17) decreased at six months compared to baseline. Additionally, there was a reduction in pain extent at one-month (F_(1.589, 52.421)_ = 8.1. *p* = 0.02. η^2^ = 0.19) and six-month follow-ups compared to baseline (F_(1.589, 52.421)_ = 8.1. *p* = 0.01. η^2^ = 0.19). The differences obtained at one month for the PPTs in the trapezius muscle did not further decrease at the six month follow-up (Trapezius 1: F_(1.421, 38.372)_ = 13.7. *p* = 1; Trapezius 2: F_(1.272, 34.354)_ = 13.48. *p* = 1; Trapezius 3: F_(1.451, 39.184)_ = 9.51. *p* = 1).

The ANCOVA revealed that the covariable pain duration (months with symptoms at baseline) only affected pain extent at one- and six-month follow-ups (F_(1.458, 46.657)_ = 10.04, *p*= 0.01, *p* = 0.006), without any significant effect on the rest of the variables.

### 3.2. Correlation Analysis between Neck/Arm Disability and Pain Sensitivity, Pain Intensity, Pain Duration and Pain Extent along Baseline, at 1-Month and 6-Month Follow-Up in the Chronic NSNP Group (Longitudinal Study)

At baseline, moderate positive correlations were observed between NDI and maximum, mean VAS and pain extent and between QuickDASH and maximum, mean VAS, and pain extent (Table 3). Moreover, a moderate positive correlation was found between NDI and QuickDASH (Table 3). At one-month follow-up, a moderate positive correlation between NDI and QuickDASH was observed. Moreover, pain extent was correlated with NDI and QuickDASH. These correlations were also evident at six-month follow-up. However, at six-month follow-up, several positive (e.g., between NDI and QuickDASH) or negative (e.g., between QuickDASH and PPTs in the trapezius and right infraspinatus) large correlations were found. A summary of all correlations between neck/arm disability and the rest of outcomes measures in the chronic NSNP group at baseline, one month, and six month follow-up are shown in Table 3.

### 3.3. Correlation Analysis between Changes in Neck/Arm Disability, Pain Sensitivity and Pain Extent

Differences between neck disability, pain sensitivity, and pain extent were evaluated to determine if the changes over time correlate, representing the change between values obtained at baseline, one month, and six month follow-ups in the chronic NSNP group (Table 4). However, no significant correlations were found.

### 3.4. Comparison of Pain Sensitivity between the Chronic NSNP and Healthy Control Groups at Baseline (Cross-Sectional Assessments)

Significant between-group differences were found at baseline in all the PPTs measurement sites with a medium/large effect size (Table 5).

## 4. Discussion

This study found that individuals with chronic NSNP demonstrated an increase in pain sensitivity following a one-month conservative physiotherapy intervention. More specifically, these individuals showed decreased PPTs, as assessed over the trapezius muscle at one-month follow-up, and gastrocnemius and infraspinatus muscle at six-month follow-up. In contrast, pain extent decreased after treatment as assessed by digital pain drawings. The total area of pain was less at one month and six months compared to baseline. The QuickDASH scored did not change, and although the NDI scores were relatively low, there was a tendency for a reduction in neck disability at six months. Furthermore, a moderate positive correlation was found between neck/arm disability and pain intensity at baseline, whereas at six-month follow-up, large positive (e.g., between NDI and QuickDASH) and negative (e.g., between arm disability and PPTs) correlations were found. Overall, these findings revealed a complicated trajectory of pain sensitivity and pain extent over time in individuals with chronic NSNP.

### 4.1. The Course of Neck/Arm Disability, Pain Sensitivity, Pain Intensity and Pain Extent in the Chronic NSNP Group

PPTs decreased at the upper trapezius, a tendency to decrease in gastrocnemius at 1-month follow-up, and a decrease at the right infraspinatus muscle at six-month follow-up, suggesting a further increase in local (neck) and remote mechanical hyperalgesia after a conservative physiotherapy intervention. This finding would indicate that pain sensitivity became progressively worse despite the completion of a one-month physical therapy intervention. Clearly, the reduction in PPTs is contrary to the expectation that a physiotherapy intervention would improve pain in the neck in parallel with pain sensitivity, as previously observed in neck pain population [62,63]. This finding and the tendency of an improved neck disability at six months may indicate that high pain sensitivity might not be directly related with neck disability. The lack of association between pain sensitivity and neck disability is supported by evidence showing positive changes in PPTs in the neck region following physiotherapy interventions, that were not correlated with changes in neck disability index scores [64]. Therefore, high pain sensitivity may be considered as an indirect consequence of pain-related factors such as poor sleep rather than a direct cause of disability [65,66].

After completion of the one-month conservative physiotherapy intervention there was a reduction in the area of pain immediately thereafter and at six months. These results may indicate that intervention influenced pain distribution. However, the association found between pain extent and pain duration suggests that the duration of symptoms may influence these changes in people with chronic NSNP. Unfortunately, the present study was not sufficiently resourced to describe a more detailed relationship between these two variables in order to draw firm conclusions. However, based on the results of our study, it seems that individuals with chronic NSNP with shorter pain duration might present more extended pain areas.

Few studies have previously investigated the relationship between disability and pain duration in individuals with spinal pain disorders [67,68]. These studies have shown that if pain persists less than one month, the likelihood of improving disability following a physiotherapy treatment is higher, whereas if pain persists more than six months, the likelihood is lower. The individuals with chronic NSNP of this study had a median pain duration of 48 months and despite the observed tendency of an improved neck function, no significant changes were observed in neck disability at six-month follow-up which is thus in accordance with previous research [67,68]. Nevertheless, it could be hypothesized that pain duration at baseline may have influenced the current neck disability results. Pain duration was investigated as a co-variable, obtaining the same results for neck disability as those derived from the analysis without pain duration as a co-variable, suggesting that once the pain condition has become chronic, the greater pain duration is no longer associated with higher disability levels. Therefore, as pain duration does not appear to directly relate to the improvement of neck disability associated to chronic NSNP, other factors (e.g., psychological factors) need to be studied in longitudinal designs in order to understand their influence on response to physiotherapy interventions.

### 4.2. Correlations between Neck/Arm Disability, Pain Sensitivity, Pain Duration and Pain Extent along Baseline, at 1-Month and 6-Months in the Chronic NSNP Group (Longitudinal Study)

At one- and six-month follow-ups, the correlation observed at baseline between the NDI and QuickDASH was maintained in the chronic NSNP group. This finding suggests that a change in neck disability directly impacts upper limb function, which is in line with another study where using the QuickDASH as an add-on to assess the impact of neck pain on upper extremity function was recommended [69]. Additionally, positive correlations were found at baseline between neck/arm disability and pain intensity as reported elsewhere [21,70,71]. Nevertheless, the present results showed a positive correlation between mean and maximum VAS and arm disability (QuickDASH) at one- and six-month follow-ups. Despite some of the QuickDASH items are directly related to pain intensity and could therefore partially explain this correlation, this finding might also indicate that pain intensity is more related with upper limb disability than it is with neck disability, which concurs with conclusions from previous literature [5].

There was no significant correlation between neck disability and pain sensitivity at any time point in the present study, which is in line with previous literature [20,21,22]. This lack of correlation may indicate that the ability of PPTs and TS to predict the severity of disability in individuals with chronic NSNP is limited. However, at six-month follow-up, a negative correlation between QuickDASH and PPTs sites in the upper trapezius and the infraspinatus muscle was found. Although no previous research have investigated this relationship specifically, one similar study conducted by Martin-Martín et al. found that arm disability was negatively correlated with PPTs [72]. Furthermore, a recent systematic review and meta-analysis found that baseline mechanical threshold modalities of QST and TS were predictive of follow-up disability and pain, respectively, for a range of musculoskeletal pain conditions such as whiplash associated disorders and low back pain [19]. There could be several explanations for these contradictory findings. First, most of the sample (82%) were females, who have demonstrated higher pain sensitivity than males [73,74], which may have influenced current results. Second, other factors such as catastrophizing, sleep quality or anxiety may also have influenced pain sensitivity in individuals with chronic NSNP as demonstrated by some authors [75,76]. Finally, assuming that altered central pain processing clinically manifests by high pain sensitivity, the present study would indicate that altered pain processing does not play a significant role in neck/arm disability in individuals with chronic NSNP.

Regarding the relationship between neck/arm disability and pain extent, the present study found a significant correlation between these variables. These findings agree with previous reports where moderate correlations between pain extent with NDI [27] and QuickDASH [77] were observed, although using cross-sectional designs.

### 4.3. Correlations over Time between Changes in Neck/Arm Disability and the Rest of Outcomes Measures in the Chronic NSNP Group (Longitudinal Study)

The present study did not show any significant association between changes in disability and changes in pain PPTs, TS and pain extent in individuals with chronic NSNP as demonstrated by the change values. This lack of correlations over time may be related to the fact that longitudinal changes in the abovementioned variables were neither significant, except pain extent. Even so, to the authors’ knowledge, this is the first study which have investigated longitudinal changes in correlation analysis in individuals with chronic NSNP. Further research is necessary for clearer conclusions.

### 4.4. Comparison in Pain Sensitivity between Chronic NSNP and Healthy Control Groups at Baseline (Cross-Sectional Study)

In line with previous cross-sectional studies [78,79], this study showed that individuals with chronic NSNP displayed a pain sensitization profile based on the presence of widespread mechanical hyperalgesia in comparison to controls, which is not influenced by the duration of symptoms. The presence of generalized mechanical hyperalgesia found in the present study in individuals with chronic NSNP may reflect the presence of altered central pain processing mechanisms and is in line with previous research done in neck pain populations [26,80]. However, contrary to our results, higher TS but not lower PPTs in comparison to healthy controls have been previously reported in individuals with chronic NSNP [16]. It is worth remarking that discordant results with TS and PPTs represent a normal finding in the literature [20,81,82], which is generalizable to other QST-modalities and leads to the convenience of implementing multimodal QST-assessment procedures in individuals with chronic musculoskeletal pain [22,83,84].

This study had also some limitations, such as the reduced sample size that does not allow to generalize results or the lack of MRI or X-ray tests, although controversy surrounds the relationship between the aforementioned tests or postural changes with pain.

Regarding the clinical implications, given the disparity across the different variable results, clinicians are recommended to incorporate tests for basic QST assessment of chronic NSNP patients, as previous research has suggested [85]. Furthermore, some variables may indicate a worsening of the condition (e.g., lower PPTs) while others may indicate an improvement (reduction in pain extent) which suggests the implementation of multimodal assessments. Finally, one of the reasons explaining the worsening or maintenance of some variables, such as PPTs or pain intensity, may be the standardized intervention, as it has been previously suggested in studies investigating physiotherapy treatments that one size does not fit all.

In conclusion, this is the first study using both cross-sectional and longitudinal designs to have assessed neck/arm disability, pain sensitivity, pain intensity, pain duration, and pain extent in individuals with chronic NSNP. The present findings indicate that pain sensitivity may worsen following a one-month conservative physiotherapy intervention despite unchanged neck/arm disability and pain intensity scores over a six-month period in individuals with chronic NSNP. However, the reduction in pain extent and its correlation with disability could indicate an improvement at one month and at six months. Studies with larger cohorts followed over a longer duration are needed to investigate the predictive value of the variables investigated in this study in individuals with NSNP.

## Figures and Tables

**Table 1:** Demographics of participants in the chronic Nonspecific Neck Pain (NSNP) and healthy control groups.

	Chronic NSNP Group (n = 34)	Healthy Control Group (n = 34)	*p*-Value
	Mean ± SD	Mean ± SD	
Age (years)	47.9 ± 10.8	44.4 ± 10.7	0.18
Gender, n female (%) ^a^	82.4%	76.5%	0.55
Height (cm)	165 ± 8.02	168.2 ± 8.7	0.30
Weight (kg)	67.9 ± 13.7	71.1 ± 12.0	0.19
Pain duration (months)	48 (24–129)		

Continuous variables are expressed as a mean and standard deviation (±SD) and categorical variables ^a^ are expressed as mean and percentage. Pain duration is expressed as a median and interquartile range (IQR) and refers to the total time with symptoms at baseline.

**Table 2:** Neck Disability Index (NDI), pain duration (months), mean/maximum Visual Analogue Scale (VAS), pain extent (pixels), Temporal Summation (TS) and Pressure Pain Thresholds (PPTs) in the chronic Nonspecific Neck Pain (NSNP) group at baseline (n = 34), 1-month (n = 34) and 6-month (n = 34) follow-up are expressed as a mean and standard deviation (±SD).

	Baseline (n = 34) Mean ± SD	1-Month (n = 34) Mean ± SD	6-Months (n = 34) Mean ± SD	Differences Baseline/1-Month (*p*-Value)	Differences 1-Month/6-Months (*p*-Value)	Differences Baseline/6-Months (*p*-Value)
NDI (0–50)	15 ± 6.2	13.65 ± 5.8	12.9 ± 6.8	0.15	1	0.05
QuickDASH (0–100)	29.9 ± 15.4	29.6 ± 15.3	31 ± 18.1	1	1	1
Maximum VAS (0–10)	7.9 ± 1.7	7.6 ± 1.5	7.7 ± 1.5	1	1	1
Mean VAS (0–10)	5.9 ± 1.5	5.7 ± 1.5	5.8 ± 1.9	1	1	1
Pain extent (0–191,246)	12,281 ± 13,837	7825 ± 10,365	7025 ± 7121	0.02 *	1	0.005 **
TS	1.7 ± 1.3	1.7 ± 1.2	1.6 ± 1	1	1	1
PPTs (kPa/cm^2^)						
Trapezius	213 ± 115	157 ± 83	151 ± 80	0.001 **	1	0.003 **
Right Infraspinatus	182 ± 98	163 ± 85	154 ± 80	0.26	0.75	0.15
Left Infraspinatus	180 ± 103	157 ± 74	145 ± 69	0.25	0.18	0.12
Gastrocnemius	230 ± 130	204 ± 90	176 ± 78	0.12	0.15	0.04 *

Repeated Measures ANOVA. * *p* < 0.05 ** *p* < 0.01. Infraspinatus 1, 2 and 3 correspond to the following sites: (1) immediately lateral to the midpoint of the medial border of the scapula; (2) at the cut-point of three lines coming from the medial point of the scapular spine, the inferior angle of the scapula, and the midpoint of the medial border of the scapula; and (3) 2 cm over the inferior angle of the scapula. Trapezius 1, 2 and 3 correspond to the following sites: (1) 2 cm lateral to the spinous process of C7; (2) midway between the spinous process of C7 and the acromion and (3) 2 cm lateral to the acromion.

**Table 3:** Pearson’s correlation coefficients (*p* values) between Neck Disability Index (NDI) and QuickDASH and the rest of outcomes in the chronic Nonspecific Neck Pain (NSNP) group.

	Baseline (n = 34)		1-Month (n = 34)		6-Months (n = 34)	
	NDI	Quick DASH	NDI	Quick DASH	NDI	Quick DASH
Pain duration	0.06 (0.70)	−0.05 (0.76)	0.2 (0.25)	0.23 (0.17)	0.01 (0.91)	−0.13 (0.43)
Maximum VAS	0.44 ** (0.008)	0.4 * (0.01)	0.23 (0.17)	0.28 (0.1)	0.37 (0.06)	0.29 (0.09)
Mean VAS	0.41 * (0.01)	0.43 *(0.01)	0.32 (0.06)	0.33 (0.06)	0.31 (0.06)	0.35 (0.06)
QuickDASH	0.58 ** (0.0)	—	0.43 ** (0.01)	—	0.73 ** (0.00)	—
Pain extent	0.57 ** (0.00)	0.36 * (0.03)	0.44 ** (0.009)	0.49 ** (0.003)	0.6 ** (0.00)	0.45 ** (0.007)
TS	−0.14 (0.41)	0.01 (0.93)	−0.08 (0.65)	−0.12 (0.49)	−0.28 (0.13)	−0.25 (0.19)
PPTs						
Trapezius	−0.04 (0.79)	0 (0.99)	−0.06 (0.73)	−0.05 (0.77)	−0.16 (0.36)	−0.30 * (0.04)
Right Infraspinatus	−0.02 (0.88)	−0.1 (0.57)	−0.14 (0.42)	−0.12 (0.47)	−0.12 (0.46)	−0.34 * (0.04)
Left Infraspinatus	0.12 (0.46)	0.03 (0.85)	−0.15 (0.38)	−0.12 (0.48)	−0.17 (0.32)	−0.34 * (0.04)
Gastrocnemius	0.08 (0.64)	0.04 (0.78)	−0.09 (0.58)	0.26 (0.12)	−0.17 (0.33)	−0.22 (0.19)

Abbreviations: Neck Disability Index (NDI), Pressure Pain Thresholds (PPTs), Temporal summation (TS), Visual Analogue Scale (VAS). Infraspinatus 1, 2 and 3 correspond to the following sites: (1) immediately lateral to the midpoint of the medial border of the scapula; (2) at the cut-point of three lines coming from the medial point of the scapular spine, the inferior angle of the scapula, and the midpoint of the medial border of the scapula; and (3) 2 cm over the inferior angle of the scapula. Trapezius 1, 2 and 3 correspond to the following sites: (1) 2 cm lateral to the spinous process of C7; (2) midway between the spinous process of C7 and the acromion and (3) 2 cm lateral to the acromion. Effect size is expressed using Pearson’s correlation coefficients (*p* values) * *p* < 0.05 ** *p* < 0.01.

**Table 4:** The difference (Δ) in neck disability (NDI), Pressure Pain Thresholds (PPTs), pain extent (pixels), in the chronic Nonspecific Neck Pain (NSNP) group between baseline and 1 and 6-months, expressed as mean and standard deviation (±SD).

	Baseline-1 Month Δ Values (n = 34)		Baseline-6 Months Δ Values (n = 34)	
Changes	Mean ± SD	NDI Index	Mean ± SD	NDI Index
NDI (0–50)	1.5 ± 4	—	2 ± 4.2	—
Pain extent (0–191,246)	4455 ± 9333	0.07 (0.68)	5255 ± 8919	0.14 (0.41)
PPTs (kPa/cm^2^)				
Trapezius	−61 ± 81	0.02 (0.88)	−61.8 ± 100	0.18 (0.3)
Gastrocnemius	−29 ± 75	−0.10 (0.58)	−55 ± 129	0.09 (0.59)

Pearson’s correlation coefficients between changes (*p*-values) in these variables along 1-month (n = 34 and 6-month (n = 34) follow-up in the chronic NSNP group. Effect size is expressed using Pearson’s correlation coefficients (*p* values). Abbreviations: Neck Disability Index (NDI), Pressure Pain Thresholds (PPTs). Infraspinatus 2 corresponds to the point located at the cut-point of three lines coming from the medial point of the scapular spine, the inferior angle of the scapula, and the midpoint of the medial border of the scapula. Trapezius 1, 2 and 3 correspond to the following sites: (1) 2 cm lateral to the spinous process of C7; (2) midway between the spinous process of C7 and the acromion and 3) 2 cm lateral to the acromion.

**Table 5:** Temporal summation (TS) and Pressure Pain Thresholds (PPTs) for the chronic Nonspecific Neck Pain (NSNP) and healthy control groups are expressed as a mean and standard deviation (±SD).

PPT-Site (kPa/cm^2^)	Mean ± SD Chronic NSNP Group (n= 34)	Mean ± SD Healthy Control Group (n = 34)	PPT Differences (*p*-Value) ^a^	Effect Size ^b^
Trapezius	213 ± 115	286 ± 121	0.011 *	0.61
Right Infraspinatus	182 ± 98	289 ± 135	0.001 **	0.91
Left Infraspinatus	180 ± 103	292 ± 136	0.001 **	0.94
Gastrocnemius	230 ± 130	319 ± 174	0.02 *	0.57
TS	1.71 ± 1.37	1.7 ± 1.4	0.98	-

^a^ The Student t test. ^b^ The d Cohen (2t/√gl). * *p* < 0.05; ** *p* < 0.01. Infraspinatus 1, 2 and 3 correspond to the following sites: (1) immediately lateral to the midpoint of the medial border of the scapula; (2) at the cut-point of three lines coming from the medial point of the scapular spine, the inferior angle of the scapula, and the midpoint of the medial border of the scapula; and (3) 2 cm over the inferior angle of the scapula. Trapezius 1, 2 and 3 correspond to the following sites: (1) 2 cm lateral to the spinous process of C7; (2) midway between the spinous process of C7 and the acromion and (3) 2 cm lateral to the acromion.

## Data Availability

The data presented in this study are available on request from the first author (GO) gortego@usj.es.
